# Omadacycline Pharmacokinetics: Influence of Mortality Risk Score among Patients with Community-Acquired Bacterial Pneumonia

**DOI:** 10.1128/aac.02201-21

**Published:** 2022-12-19

**Authors:** M. Trang, J. P. Hammel, E. A. Lakota, M. C. Safir, S. M. Bhavnani, L. Friedrich, J. N. Steenbergen, P. C. McGovern, E. Tzanis, C. M. Rubino

**Affiliations:** a Institute for Clinical Pharmacodynamics, Inc., Schenectady, New York, USA; b Paratek Pharmaceuticals, Inc., King of Prussia, Pennsylvania, USA

**Keywords:** omadacycline, pharmacokinetics, community-acquired bacterial pneumonia

## LETTER

Omadacycline is approved in the United States to treat patients with community-acquired bacterial pneumonia (CABP) (1). In the phase 3 OPTIC study, a difference in mortality between omadacycline- and moxifloxacin-treated patients with CABP was noted (2). However, subsequent analyses found no differences between treatment groups to explain this mortality imbalance (3). The assessment of an exposure-response relationship for mortality among omadacycline-treated patients was not possible given few deaths (8 of 386) and that measured omadacycline concentrations were available for only 50 patients, none of whom died. However, to understand whether the risk of mortality is associated with omadacycline exposure, the influence of the baseline Pneumonia Patient Outcomes Research Team (PORT) risk class (4) and CURB-65 score (based on confusion, urea, respiratory rate, blood pressure, and age of ≥65) (5) on the pharmacokinetics (PK) of omadacycline for the 50 patients was evaluated.

**FIG 1:**
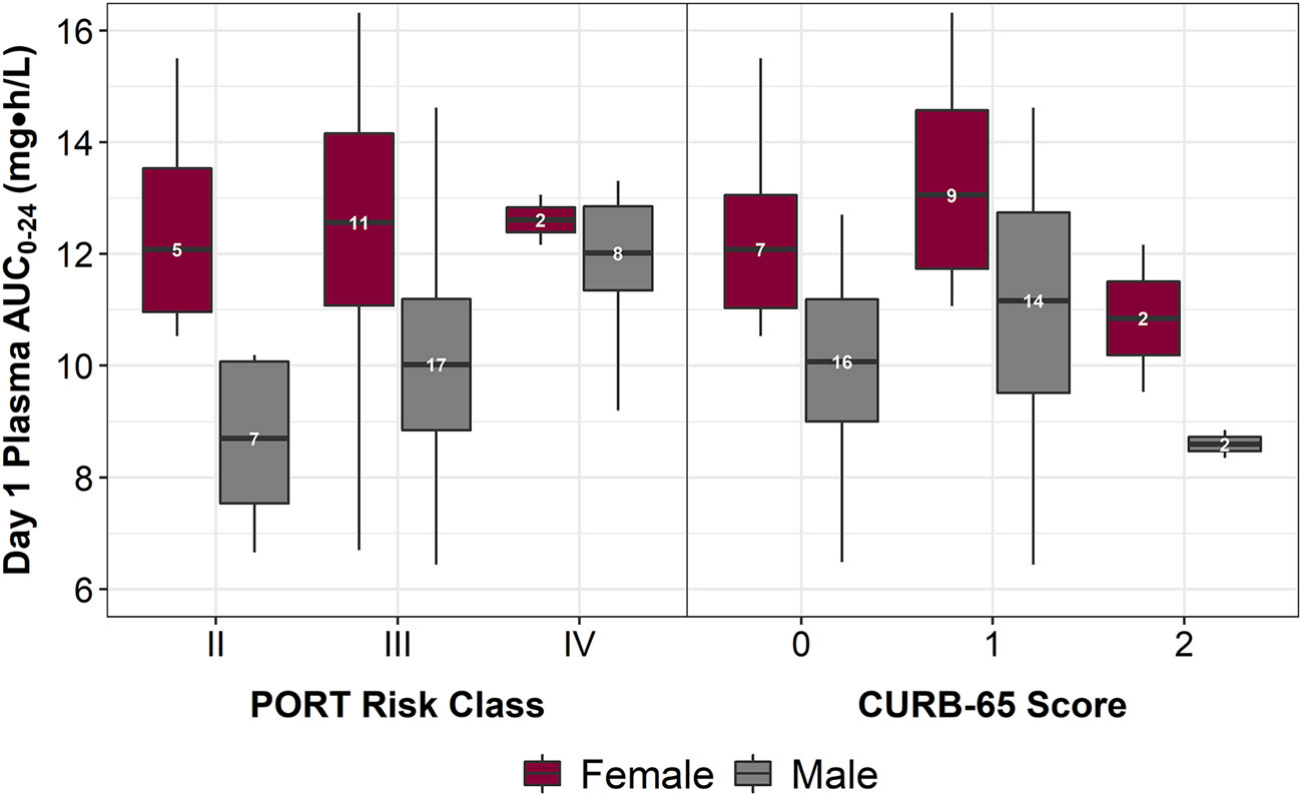
Box-and-whisker plot showing the distribution of day 1 omadacycline total-drug plasma AUC_0–24_, stratified by sex and each PORT risk class and CURB-65 score. The upper and lower edges of each box represent the 25th and 75th percentiles of the distribution of total-drug plasma AUC_0–24_, respectively. The line within each box represents the median total-drug plasma AUC_0–24_ value. The upper and lower whiskers extend to the furthest observed values within 1.5 × IQR of the respective box edges (where IQR is defined as the difference between the 25^th^ and 75^th^ percentiles).

Summaries of baseline patient descriptors, stratified by the risk scores, are presented for the above-described 50 patients in Table S1 in the supplemental material. Table 1 shows the mean (standard deviation [SD]) day 1 omadacycline total-drug plasma AUC_0–24_ (area under the concentration-time curve from 0 to 24 h) values by the risk scores. Given that the results of a previous covariate analysis showed that females have significantly slower clearance than males (6), box-and-whisker plots showing the distributions of AUC_0–24_ stratified by sex and each risk score are provided in Fig. 1.

**TABLE 1:** Omadacycline total-drug plasma AUC_0–24_ by PORT risk class and CURB-65 score

Stratification variable	*n*	Mean (SD) day 1 total-drug plasma AUC_0–24_ (mg·h/L)	Estimated mean (95% Cl) sex adjusted group difference in AUC_0–24_^*a*^	
			Comparisons relative to PORT risk class II or CURB-65 score of 0	Comparisons relative to PORT risk class III or CURB-65 score of 1
PORT risk class				
II	12	10.3 (2.57)		
III	28	11.0 (2.56)	0.778 (−0.681, 2.24)	
IV	10	12.0 (1.26)	2.22 (0.388, 4.05)	1.44 (−0.135, 3.02)
CURB-65 score				
0	23	10.6 (2.14)		
1	23	11.6 (2.62)	0.804 (−0.472, 2.08)	
2	4	9.72 (1.70)	−1.34 (−3.68, 1.01)	−2.14 (−4.48, 0.200)

a Based on a two-way analysis of variance (ANOVA) model.

Two-way analysis of variance (ANOVA), adjusting for sex, was performed to assess the relationships between AUC_0–24_ and each risk score. Based on the models summarized in Table S2, the mean AUC_0–24_ values were lower for males than females (*P* ≤ 0.002), and there was evidence of a sex-adjusted difference in the mean AUC_0–24_ values between PORT risk classes II and IV (*P* = 0.019). Interactions between sex and each risk score were investigated but were not statistically significant (*P* ≥ 0.287) and were not retained in the models.

Estimates for the sex-adjusted pairwise group differences in the mean AUC_0–24_ based on the two-way ANOVA are provided in Table 1. Based on the 95% confidence intervals, the largest estimated magnitudes were no more than 39.3 and 46.1% of the group means for PORT risk class and CURB-65 score, respectively. Since the AUC/MIC ratio is the pharmacokinetic-pharmacodynamic index that predicts efficacy for omadacycline, differences in the mean AUC_0–24_ of at least halving or doubling are required to account for a full dilution change in the MIC when evaluating the probability of achieving AUC/MIC ratio targets associated with a 1-log_10_ CFU reduction from baseline for Streptococcus pneumoniae or Haemophilus influenzae for the treatment of patients with CABP (7). In light of these considerations, the magnitude of differences based on the risk scores was not considered clinically relevant.

The number of patients with measured omadacycline concentrations (50/386) was one limitation of these analyses. Compared to the remaining patients in the intent-to-treat population, this subset was younger with a higher mean baseline creatinine clearance and a reduced history of hypertension. No other statistically significant differences were found, including those based on sex, PORT risk class, and CURB-65 score. Given this limitation, further PK assessments among omadacycline-treated patients with CABP should be carried out to confirm the findings described herein.

These findings suggest that the observed mortality imbalance was not due to differences in omadacycline PK.
