# Supplementary material for: Omadacycline Pharmacokinetics: Influence of Mortality Risk Score among Patients with Community-Acquired Bacterial Pneumonia
Source: Antimicrob Agents Chemother. 2022 Dec 19;67(1):e02201-21. doi: 10.1128/aac.02201-21 (PMC9872632; doi:10.1128/aac.02201-21)
Supplement: Supplemental file 1 — Supplemental material. Download aac.02201-21-s0001.pdf, PDF file, 0.09 MB [file aac.02201-21-s0001.pdf]

Supplemental Table 1. Summary statistics for the demographics of the PK analysis population and stratified by PORT Risk Class and CURB-65

Variable	All patients (N=50)	PORT Risk Class			CURB-65 score		
		II (N=12)	III (N=28)	IV (N=10)	0 (N=23)	1 (N=23)	2 (N=4)
Age (yr)	54.0 (28.0, 84.0)	51.5 (28.0, 63.0)	54.0 (31.0, 79.0)	69.0 (49.0, 84.0)	55.0 (28.0, 63.0)	54.0 (31.0, 84.0)	49.5 (31.0, 75.0)
Weight (kg)	77.5 (36.0,127)	78.0 (58.5, 127)	77.5 (36.0, 97.0)	77.0 (50.0, 95.0)	82.0 (58.5, 127)	72.0 (36.0, 101)	83.5 (81.0, 86.0)
Height (cm)	174 (137,192)	175 (137, 188)	174 (150, 192)	168 (150, 180)	176 (137, 190)	167 (150, 184)	171 (168, 192)
BSA (m^2)	1.91 (1.25, 2.38)	1.96 (1.44, 2.38)	1.90 (1.25, 2.22)	1.86 (1.43, 2.13)	2.02 (1.44, 2.38)	1.87 (1.25, 2.17)	1.98 (1.92, 2.10)
BMI (kg/m^2)	26.2 (16.0, 41.5)	29.3 (20.4, 41.5)	24.3 (16.0, 31.3)	27.4 (20.0, 31.2)	25.3 (20.0, 41.5)	26.4 (16.0, 32.6)	28.7 (22.0, 30.1)
CLcr (mL/min/1.73 m^2)	85.2 (33.1, 162)	81.0 (45.8, 145)	91.7 (55.1, 162)	51.3 (33.1, 115)	85.3 (45.8, 162)	85.2 (33.1, 145)	83.7 (68.7, 126)
Albumin (mg/dL)	4.05 (2.70, 5.00)	3.90 (3.60, 4.50)	4.30 (3.10, 5.00)	3.90 (2.70, 4.50)	4.10 (3.30, 5.00)	4.00 (2.70, 5.00)	4.30 (3.20, 4.80)
Sex							
Male	32/50 (64.0)	7/12 (58.3)	17/28 (60.7)	8/10 (80.0)	16/23 (69.6)	14/23 (60.9)	2/4 (50.0)
Female	18/50 (36.0)	5/12 (41.7)	11/28 (39.3)	2/10 (20.0)	7/23 (30.4)	9/23 (39.1)	2/4 (50.0)

Note: All values are presented as median (minimum, maximum) except for sex, which is presented as n/N (Percent). BMI, body mass index; BSA, body surface area; CLcr, creatinine clearance.

Supplemental Table 2. Results of two-way ANOVA analyses for Day 1 omadacycline total-drug plasma $AUC_{0\text{-}24}$ accounting for PORT Risk Class/CURB-65 score and sex

Category	Parameter Estimate	95% CI	p-value[a]
Base case (males; PORT Risk Class of II)	9.31	7.99, 10.6	
Covariate effects:			
PORT Risk Class of III	0.778	-0.681, 2.24	0.289
PORT Risk Class of IV	2.22	0.388, 4.05	0.019
Females	2.32	1.06, 3.58	0.0006
Base case (males; CURB-65 score of 0)	10.0	(9.03, 11.0)	
Covariate effects:			
CURB-65 score of 1	0.804	-0.472, 2.08	0.211
CURB-65 score of 2	-1.34	-3.68, 1.01	0.258
Females	2.09	0.810, 3.37	0.002

Note: CI, confidence interval for the parameter estimate.

a. F-test from ANOVA.
